# Essay on the Disorders Incident to Literary Men

**Published:** 1837-01

**Authors:** 


ART. I.—*Essay on the Disorders incident to Literary Men; and on the best Means of preserving their Health. Read before the Royal Society of Literature.* By W. NEWNHAM, Esq.—*London*, 1836. 8vo. pp. 36.

WE have no hesitation in saying that this little work contains, in our opinion, more matter of real value than all the numerous volumes which have preceded it on the same subject. For the first time, the diseases peculiar to men of letters have been viewed in their true light in this pamphlet, as depending essentially on a disordered state of the organ of the mind, the brain; and the various disorders which have been commonly set down to sedentariness or want of bodily exercise, and other analogous causes, are here traced to their true source, over-exertion of the brain in the elaboration of thought. We have long been convinced,—and the experience of every additional year strengthens the conviction,—that by much the most frequent cause of dyspepsia, in all its forms, is mental disorder, —mental distress, anxiety, irritation, or exhaustion; and we had been led by observation, and we may be allowed to add, by personal experience, to adopt the very same views respecting the prevailing sources of the diseases of literary men, and we would say of men of business generally, which are so admirably developed in the present essay by Mr. Newnham. What had heretofore been treated merely empirically or on false and hypothetical grounds, Mr. Newnham has brought within the domain of rational pathology; and, if there may remain doubts as to some of his explanations of the *modus operandi* of the causes, and as to the exact pathological condition of the parts affected, no impartial enquirer will any more doubt of the brain being the primary seat of the disorders in question, than he will doubt of the lungs being the seat of pneumonia, or the liver of hepatitis.

A great step has béen made of late years towards a rational pathology of nervous diseases generally, and what have been commonly termed mental diseases, by the general recognition of the great nervous centres as their site; and it cannot be denied that for much of this improvement we are indebted to the investigations and enquiries of phrenology. Whatever opinions are held respecting the ultimate cause of the mental phenomena, no physiologist now doubts that the brain is their essential seat, and as necessary for their development and manifestation as the stomach is for digestion, or the kidney for the secretion of urine. It is therefore just as reasonable to expect that the organ which is over-exerted in the labours of the study should be the one principally to suffer in the student, as that any organ which is especially exposed to morbid influences in the artisan should be particularly obnoxious to disease in him. And yet, as we have already hinted, this simple and obvious truth was never clearly and distinctly exposed, in this country at least, before the publication of Mr. Newnham's: certainly it was never submitted to the profession in a form so well calculated to convey just views and to lead to practical results of the greatest importance. In our next Number we shall prosecute the same subject, in noticing the valuable works of Dr. Brigham.

Mr. Newnham lays claim to no originality: still, if it be a merit to collect into one focus the rays of scattered light, and direct them to the clearing up of theoretical difficulties, and to the institution of beneficial practical processes, to that merit our author is certainly entitled. We recommend the work to the particular attention of such of our junior brethren as are hard students; it is fraught with warnings which they will do well to attend to in time; and we recommend it to all who have for their patients the victims of an over-excited and over-tasked brain, whether the field of labour be the study or the counting-house. To such persons their medical attendant can give no more valuable prescription than the perusal of Mr. Newnham's pamphlet, as it cannot fail to come strongly in aid of the only rational treatment to be instituted in such cases,—abatement or entire abandonment of the practices which have produced and still keep up the disease.

The literary execution of this little work is such as might be expected from a gentleman of the acquirements and habits of its author; but we must protest against the use of the word *brainular,* which meets our eye in many pages, and which cannot be defended on any grounds, whether of necessity, etymology, or taste. Unwilling as we are to countenance words of foreign origin where we possess already a good native synonym, the attempt to introduce such a pseudo-English term as the one in question is altogether preposterous, and when we have the word *cerebral,* now for a long time familiarized to us, in the medical language of this country.

